# Associations between Patient Global Assessment scores and pain, physical function, and fatigue in rheumatoid arthritis: a post hoc analysis of data from phase 3 trials of tofacitinib

**DOI:** 10.1186/s13075-020-02324-7

**Published:** 2020-10-15

**Authors:** Vibeke Strand, Jeffrey Kaine, Rieke Alten, Gene Wallenstein, Annette Diehl, Harry Shi, Rebecca Germino, Christopher W. Murray

**Affiliations:** 1 Division of Immunology/Rheumatology, Stanford University, Palo Alto, CA USA; 2 Independent Healthcare Associates Inc, Cullowhee, NC USA; 3 Schlosspark-Klinik, University Medicine, Berlin, Germany; 4 Pfizer Inc, Groton, CT USA; 5 Pfizer Inc, Collegeville, PA USA; 6 Pfizer Inc, New York, NY USA

**Keywords:** Disability, Fatigue, Pain, Patient Global Assessment, Patient-reported outcomes, Physical function, Rheumatoid arthritis, Tofacitinib

## Abstract

**Background:**

Tofacitinib is an oral Janus kinase inhibitor for the treatment of rheumatoid arthritis (RA). We examined the degree to which Patient Global Assessment of Disease Activity (PtGA) was driven by patient-reported assessments of pain (Pain), physical function, and fatigue in patients receiving tofacitinib 5 mg twice daily or placebo, each with conventional synthetic disease-modifying antirheumatic drugs (csDMARDs).

**Methods:**

This post hoc analysis used data pooled from three randomized controlled trials in csDMARD-inadequate responder (csDMARD-IR) patients (ORAL Scan: NCT00847613; ORAL Standard: NCT00853385; ORAL Sync: NCT00856544). Using subgroup analysis from 2 × 2 tables, associations between PtGA and Pain, Health Assessment Questionnaire-Disability Index (HAQ-DI), and Functional Assessment of Chronic Illness Therapy-Fatigue (FACIT-F) at month 3 were evaluated using Pearson’s Phi correlation coefficients. To support the main analysis, associations between select patient-reported outcomes (PROs) were also evaluated in csDMARD-naïve (ORAL Start; NCT01039688) and biologic (b)DMARD-IR (ORAL Step; NCT00960440) patients.

**Results:**

Across csDMARD-IR treatment groups, low disease activity (defined as PtGA ≤ 20 mm), and moderate (≥ 30%) and substantial (≥ 50%) improvements from baseline in PtGA were associated with mild Pain (Visual Analog Scale score ≤ 20 mm), and moderate (≥ 30%) and substantial (≥ 50%) improvements from baseline in Pain; lack of Pain improvement was associated with little/no improvement in PtGA. In contrast, large proportions of csDMARD-IR patients who reported PtGA improvements did not report HAQ-DI or FACIT-F scores ≥ normative values (≤ 0.25 and ≥ 43.5, respectively) or changes in HAQ-DI or FACIT-F scores ≥ minimum clinically important difference (≥ 0.22 and ≥ 4.0, respectively). Generally, PtGA and Pain outcomes were moderately-to-strongly correlated at month 3 in csDMARD-IR patients, with weaker correlations evident between PtGA and HAQ-DI/FACIT-F outcomes. Similar findings were generally evident in csDMARD-naïve and bDMARD-IR patients.

**Conclusions:**

This analysis supports the role of Pain as a key driver of PtGA in RA; physical function and fatigue play lesser roles in patients’ perceptions of disease activity. These findings corroborate the importance of improved PROs and attainment of low symptom states for optimizing patient care.

**Trial registration:**

Clinicaltrials.gov: NCT00847613 (registered: February 19, 2009); NCT00853385 (registered: March 2, 2009); NCT00856544 (registered: March 5, 2009); NCT01039688 (registered: December 25, 2009); NCT00960440 (registered: August 17, 2009)

## Background

Rheumatoid arthritis (RA) is associated with high levels of pain, impaired physical function, and diminished health-related quality of life (HRQoL) [1]. From the patient perspective, improvements in these outcomes remain a priority when evaluating RA treatment strategies [2]. Accordingly, the importance of including patient-reported outcomes (PROs) in randomized controlled trials (RCTs) has long been recognized by professional bodies such as the Outcome Measures in Rheumatology (OMERACT) international consensus effort [3, 4], American College of Rheumatology (ACR) [5], and European League Against Rheumatism (EULAR) [6], as well as the US Food and Drug Administration [7] and payers, such as the Institute for Quality and Efficiency in Healthcare in Germany [8].

One of the most widely reported PROs in RA is the Patient Global Assessment of Disease Activity (PtGA), one of the seven ACR core set components recommended for assessment in RCTs [5, 9]. Important drivers of PtGA include patient assessment of pain (Pain; Visual Analog Scale [VAS]) and physical function (measured by the Health Assessment Questionnaire-Disability Index [HAQ-DI]) [9], both of which are also patient-reported components of the ACR core set [5] known to reflect disease activity [9]. PtGA, Pain, and HAQ-DI are more sensitive to change than laboratory measures, making them a valuable measure of treatment efficacy [10]. Outside the ACR core set, fatigue is also recognized as an important measure in RA RCTs [11], and notably, has been shown to be a further key determinant of PtGA [12]. While physical function [9, 13] and fatigue [12] influence PtGA, several studies have identified Pain as the main driver of PtGA, explaining approximately 75% of the reported results [9, 12–15].

Interestingly, discordance between patient and physician assessment of RA has been reported in over a third of RA patients, with patients with discordance typically reporting a higher level of disease activity than their physicians [14, 16]. Such discrepancies have been shown to negatively impact therapeutic outcomes [9], with discordance contributing to worse HRQoL, activity impairment, and reduced work productivity [17, 18]. Pain appears to be the most important domain to patients, whereas Physician Global Assessment of Arthritis (MDGA) is driven by physician-assessed measures of swollen and tender joint counts (SJC and TJC, respectively) and levels of inflammation markers [9, 14, 17]. As patients tend to weight Pain into PtGA to a greater extent than physicians weight joint counts into MDGA [13], it is unsurprising that Pain has been shown to be a key driver of discordance [12, 13, 17, 19], with associated higher fatigue and disability scores [17].

In recent years, the Janus kinase and signal transducer and activator of transcription (JAK-STAT) pathway has been shown to play a central role in both inflammatory and neurogenic pain processes associated with RA and other autoimmune disorders [20, 21]. As such, blocking elements of JAK-STAT signaling represents an attractive therapeutic strategy [22–24].

Tofacitinib is an oral JAK inhibitor for the treatment of RA. Sustained improvements in PROs, including PtGA, Pain, HAQ-DI, and fatigue (measured using the Functional Assessment of Chronic Illness Therapy-Fatigue [FACIT-F] scale), as well as HRQoL (measured by the 36-item Short Form Health Survey [SF-36]) have previously been reported in phase 2 [25], phase 3 [26–30], and phase 3b/4 RCTs of tofacitinib [31]. In brief, tofacitinib, administered as monotherapy or combination therapy, resulted in statistically significant improvements from baseline in all five outcomes (PtGA, Pain, HAQ-DI, FACIT-F, and SF-36) versus placebo [25–27, 29, 30] or methotrexate (MTX) [28], that was maintained for the duration of tofacitinib treatment (up to 24 months). Notably, in ORAL Solo, a phase 3 placebo-controlled RCT of tofacitinib in DMARD-inadequate responder (IR) patients, benefits of treatment with tofacitinib monotherapy were shown to be rapid in onset, with significant improvements evident at week 2 in PtGA, Pain, and HAQ-DI, and changes from baseline in PtGA and Pain reported as early as day 3 (after baseline, FACIT-F and SF-36 were not measured until month 3) [26]. Furthermore, in ORAL Strategy, a phase 3b/4 head-to-head non-inferiority RCT of tofacitinib monotherapy versus tofacitinib or adalimumab in combination with MTX in MTX-IR RA patients, clinically meaningful improvements from baseline in PtGA, Pain, HAQ-DI, FACIT-F, and SF-36 were reported in all three treatment arms [31]. In long-term extension (LTE) studies, tofacitinib, with or without conventional synthetic disease-modifying antirheumatic drugs (csDMARDs; mostly MTX), was associated with sustained improvements in HAQ-DI [32–34].

While improvements in PROs have been demonstrated, the associations between PtGA and Pain, between PtGA and HAQ-DI, and between PtGA and FACIT-F have not previously been investigated in RA patients treated with tofacitinib. This post hoc analysis used pooled data from three phase 3 RCTs of csDMARD-IR RA patients receiving tofacitinib in combination with csDMARDs to further examine and understand the degree to which PtGA is associated with patient-reported improvements in Pain, physical function (HAQ-DI), and fatigue (FACIT-F), and gain insights into these relationships. To support the main analysis, associations between select PROs were also evaluated in csDMARD-naïve (ORAL Start; NCT01039688) [28]) and biologic (b)DMARD-IR (ORAL Step; NCT00960440 [30]) patients.

## Methods

### Phase 3 study design

This post hoc analysis included data from three phase 3 RCTs of tofacitinib with similar designs: 12-month ORAL Sync (*n* = 795; NCT00856544) [29, 35], ORAL Standard (*n* = 717; NCT00853385) [27, 36], and 24-month ORAL Scan (*n* = 797; NCT00847613) [37, 38].

In brief, all three RCTs were conducted globally and enrolled csDMARD-IR (MTX-IR in ORAL Standard and ORAL Scan) patients (aged ≥ 18 years) with RA according to the ACR 1987 Revised Criteria [39]; active disease was defined as ≥ 6 tender and ≥ 6 swollen joints (≥ 4 for each in ORAL Sync; all evaluated using 68/66-joint count) and erythrocyte sedimentation rate (Westergren method) > 28 mm/h, or C-reactive protein (CRP) level > 7 mg/L. Tofacitinib 5 mg and 10 mg twice daily (BID) and placebo were administered in combination with csDMARDs (specifically MTX in ORAL Standard and ORAL Scan); ORAL Standard also included an active comparator arm (adalimumab 40 mg administered subcutaneously every 2 weeks). Patients receiving placebo combination therapy were advanced in a blinded manner to tofacitinib 5 mg or 10 mg BID if they had not achieved ≥ 20% improvement in SJC and TJC after 3 months (defined as non-responders); after 6 months, all remaining placebo patients were advanced to tofacitinib.

To support the main analysis conducted in csDMARD-IR patients, additional analyses were conducted using data from csDMARD-naïve and bDMARD-IR patients enrolled in the phase 3 RCTs ORAL Start (NCT01039688) and ORAL Step (NCT00960440). Full details of the study design have been reported previously [28, 30, 40, 41]. Briefly, ORAL Start was a 24-month phase 3 RCT that included csDMARD-naïve RA patients receiving tofacitinib 5 or 10 mg BID monotherapy, or MTX monotherapy (*n* = 958; NCT01039688) [28]; ORAL Step was a 6-month RCT that included bDMARD-IR RA patients receiving tofacitinib 5 or 10 mg BID, or placebo (advancing to tofacitinib 5 or 10 mg BID at month 3), all with background MTX (*n* = 399; NCT00960440) [30].

All RCTs were conducted in accordance with the Declaration of Helsinki and International Conference on Harmonization Guidelines for Good Clinical Practice and approved by the institutional review board and/or independent ethics committee for each study center. All patients provided written informed consent.

### Post hoc analysis

In this post hoc analysis, data from the three phase 3 RCTs of csDMARD-IR patients were pooled and PtGA, Pain, HAQ-DI, and FACIT-F were assessed at baseline and month 3 (last blinded-placebo controlled time point) for the tofacitinib 5 mg BID and placebo groups. PtGA and Pain were evaluated using a VAS of 0–100 mm. In the supporting analysis, PtGA, Pain, and HAQ-DI were assessed at baseline and month 3 for csDMARD-naïve patients receiving tofacitinib 5 mg BID or MTX as monotherapy, and for bDMARD-IR patients receiving tofacitinib 5 mg BID or placebo, both with background MTX.

For the purposes of the current analyses, low disease activity (LDA) was defined as PtGA score ≤ 20 mm [42], and moderate and substantial improvements in PtGA were defined as decreases from baseline of ≥ 30% and ≥ 50%, respectively [43]. Similarly, mild Pain was a score ≤ 20 mm [42], and moderate and substantial improvements in Pain were defined as decreases from baseline of ≥ 30% and ≥ 50%, respectively [44, 45]. A clinically meaningful HAQ-DI response was defined as a score of ≤ 0.25 (normative value) [46] or an improvement from baseline of ≥ 0.22 (minimum clinically important difference [MCID]) [47]. A clinically meaningful FACIT-F response was defined as a score of ≥ 43.5 (normative value; based on mean FACIT-F scores observed in two large general population studies [48, 49]) or an increase from baseline of ≥ 4.0 (MCID) [50].

### Statistical analysis

Data are presented for the full analysis set (all patients randomized to treatment who received ≥ 1 dose of study drug and had ≥ 1 post-baseline assessment).

In the main analysis, subgroup analysis from 2 × 2 tables separately evaluated the associations for PtGA and Pain, PtGA and HAQ-DI, and PtGA and FACIT-F at month 3, using the respective PRO cut-offs outlined above. In the supporting analysis, subgroup analysis from 2 × 2 tables separately evaluated the associations for PtGA and Pain (PtGA-defined LDA with mild Pain; PtGA-defined LDA with substantial [≥ 50%] improvement in Pain; substantial PtGA improvements with mild Pain), and PtGA and HAQ-DI (PtGA-defined LDA with HAQ-DI ≥ normative values).

At month 3, Pearson Phi correlation coefficients along with *P* values testing whether the coefficients were significant from 0 were calculated. The *P* values presented are not adjusted for multiplicity. Generally, correlation coefficient values around 0.3, 0.5, and 0.7 are considered as weak, moderate, and strong positive linear correlations, respectively.

## Results

### Patients

In total, 1133 csDMARD-IR patients were included in this post hoc analysis, of whom 742 received tofacitinib 5 mg BID and 391 received placebo, both in combination with csDMARDs (mostly MTX). Patient demographics and baseline disease characteristics, including PtGA, Pain, HAQ-DI, and FACIT-F scores, were generally similar across both pooled csDMARD-IR treatment groups (Table 1). Patient demographics and baseline disease characteristics of patients in the supporting analysis have been published previously [40, 41] and were generally similar among treatment groups within the csDMARD-naïve and bDMARD-IR cohorts.

**Table 1:** Demographics and baseline disease characteristics of csDMARD-IR patients included in the post hoc analysis

Demographic or baseline characteristic^**a**^	Tofacitinib 5 mg BID + csDMARDs (***N*** = 742)	Placebo + csDMARDs (***N*** = 391)
Age (years), mean (range)	53.1 (18–86)	52.4 (18–81)
Female, *n* (%)	625 (84.2)	314 (80.3)
Smoking, *n* (%)		
Never smoked	511 (68.9)	260 (66.5)
Smoker	96 (12.9)	74 (18.9)
Ex-smoker	135 (18.2)	55 (14.1)
Duration of RA (years), mean (range)	8.0 (0.2–43.0)	8.8 (0.3–49.4)
DAS28-4(ESR) score, mean (SD)	6.4 (1.0)	6.3 (1.0)
TJC, mean (SD)	25.1 (14.7)	24.7 (14.0)
SJC, mean (SD)	14.8 (9.2)	14.8 (8.7)
ESR (mm/h), mean (SD)	50.1 (25.7)	50.2 (24.6)
RF-positive, *n* (%)	630 (84.9)	325 (83.1)
PtGA VAS score (mm), mean (SD)	58.3 (23.0)	55.0 (22.5)
Pain VAS score (mm), mean (SD)	57.7 (23.0)	55.2 (22.5)
HAQ-DI score, mean (SD)	1.4 (0.7)	1.3 (0.7)
FACIT-F score, mean (SD)	29.0 (10.7)	30.6 (10.2)
SF-36 PCS score, mean (SD)	33.1 (7.9)	33.7 (7.5)
SF-36 MCS score, mean (SD)	40.6 (11.9)	42.6 (11.5)
MDGA VAS score, mean (SD)	59.2 (16.9)	58.0 (16.9)
Concomitant therapy, *n* (%)	742 (100.0)	391 (100.0)
Oral corticosteroids, *n* (%)	479 (64.6)	246 (62.9)
Non-MTX csDMARDs, *n* (%)	152 (20.5)	75 (19.2)
MTX, *n* (%)	677 (91.2)	354 (90.5)
Concomitant MTX dose (mg), mean (range)	14.3 (1.4–25.0)	14.9 (1.0–25.0)

Data presented for the full analysis set

Abbreviations: *BID* twice daily, *csDMARD* conventional synthetic disease-modifying antirheumatic drug, *DAS28-4(ESR)* Disease Activity Score in 28 joints, ESR, *ESR* erythrocyte sedimentation rate, *FACIT-F* Functional Assessment of Chronic Illness Therapy-Fatigue, *HAQ-DI* Health Assessment Questionnaire-Disability Index, *IR* inadequate responder, *MCS* Mental Component Summary, *MDGA* Physician Global Assessment of Arthritis, *MTX* methotrexate, *PCS* Physical Component Summary, *PtGA* Patient Global Assessment of Disease Activity, *RA* rheumatoid arthritis, *RF* rheumatoid factor, *SD* standard deviation, *SF-36* 36-item Short Form Health Survey, *SJC* swollen joint count, *TJC* tender joint count, *VAS* Visual Analog Scale

^a^*n* values for individual characteristics may vary

### Patient-reported outcomes at month 3

The proportions of csDMARD-IR patients reporting each clinically meaningful PRO improvement at month 3 are shown in Table 2. Across all endpoints, responses were numerically higher in patients treated with tofacitinib 5 mg BID versus placebo; these differences were particularly marked for PtGA and Pain. Over 60% of patients treated with tofacitinib 5 mg BID reported moderate improvements (≥ 30% decrease from baseline) in PtGA and Pain, with a large proportion (> 40%) also reporting substantial improvements (≥ 50% decrease from baseline) across PtGA and Pain. Approximately 35% of tofacitinib-treated patients reported a mild Pain score (VAS score ≤ 20 mm) and approximately 20% of patients reported HAQ-DI and FACIT-F scores ≥ normative values, while > 50% of tofacitinib-treated patients reported improvements in HAQ-DI and FACIT-F scores ≥ MCID.

**Table 2:** Proportion of csDMARD-IR patients reporting each clinically meaningful PRO improvement at month 3

PRO, ***n*** (%)	Tofacitinib 5 mg BID + csDMARDs (***N*** = 695)	Placebo + csDMARDs (***N*** = 366)
PtGA		
LDA (PtGA VAS score ≤ 20 mm)	216 (31.1)	62 (16.9)
Moderate PtGA improvement (≥ 30% decrease from baseline)^a^	421 (60.8)	119 (32.6)
Substantial PtGA improvement (≥ 50% decrease from baseline)^a^	301 (43.5)	73 (20.0)
Pain		
Mild Pain (VAS score ≤ 20 mm)	246 (35.4)	62 (16.9)
Moderate Pain improvement (≥ 30% decrease from baseline)^a^	419 (60.5)	124 (34.0)
Substantial Pain improvement (≥ 50% decrease from baseline)^a^	310 (44.8)	70 (19.2)
HAQ-DI		
HAQ-DI score ≥ normative value (≤ 0.25)	142 (20.4)	44 (12.0)
HAQ-DI change ≥ MCID (≥ 0.22 improvement from baseline)^b^	463 (66.8)	166 (45.6)
FACIT-F		
FACIT-F score ≥ normative value (≥ 43.5)^c^	138 (19.9)	46 (12.6)
FACIT-F change ≥ MCID (≥ 4.0 improvement from baseline)^d^	381 (55.0)	131 (36.1)

Abbreviations: *BID* twice daily, *csDMARD* conventional synthetic disease-modifying antirheumatic drug, *FACIT-F* Functional Assessment of Chronic Illness Therapy-Fatigue, *HAQ-DI* Health Assessment Questionnaire-Disability Index, *IR* inadequate responder, *LDA* low disease activity, *MCID* minimum clinically important difference, *PRO* patient-reported outcome, *PtGA* Patient Global Assessment of Disease Activity, *VAS* Visual Analog Scale

^a^Tofacitinib 5 mg BID + csDMARDs, *N* = 692; placebo + csDMARDs, *N* = 365

^b^Tofacitinib 5 mg BID + csDMARDs, *N* = 693; placebo + csDMARDs, *N* = 364

^c^Tofacitinib 5 mg BID + csDMARDs, *N* = 694; placebo + csDMARDs, *N* = 365

^d^Tofacitinib 5 mg BID + csDMARDs, *N* = 693; placebo + csDMARDs, *N* = 363

The proportions of csDMARD-naïve and bDMARD-IR patients reporting each clinically meaningful PRO improvement at month 3 are shown in Supplementary Table 1. Responses were generally numerically higher in csDMARD-naïve patients receiving tofacitinib 5 mg BID monotherapy versus MTX monotherapy, and in bDMARD-IR patients receiving tofacitinib 5 mg BID with MTX versus placebo with MTX. In tofacitinib-treated csDMARD-naïve patients, approximately 46% of patients reported substantial improvements (≥ 50% decrease from baseline) in PtGA, 50% reported substantial improvements in Pain, and approximately 30% reported HAQ-DI scores ≥ normative values. In tofacitinib-treated bDMARD-IR patients, approximately 38% and 44% of patients reported substantial improvements in PtGA and Pain, respectively, and 16% reported HAQ-DI scores ≥ normative values.

### Associations between patient-reported outcomes at month 3

**Fig. 1:**
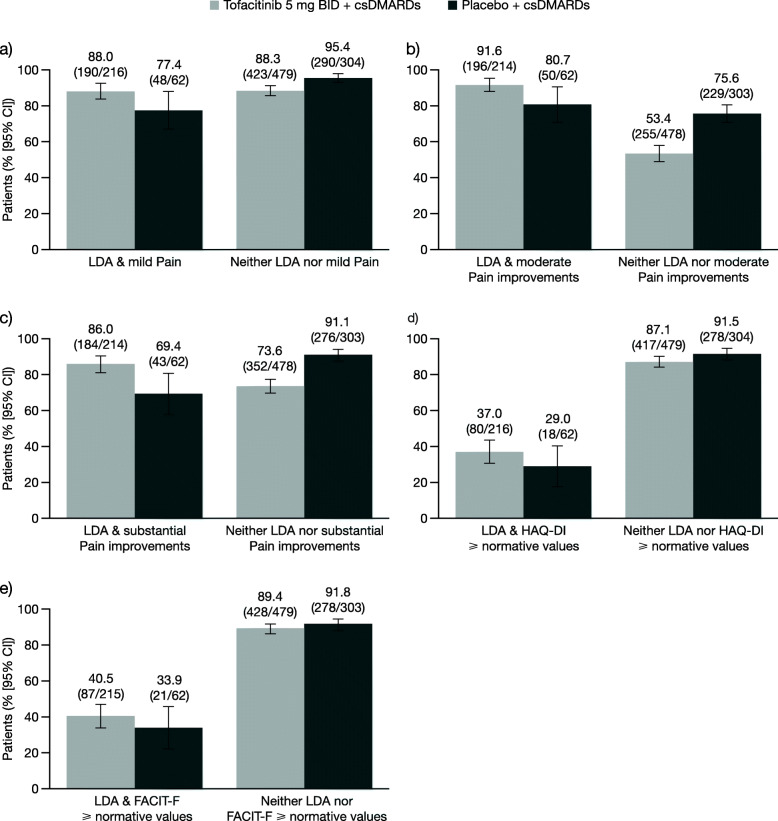
Associations between LDA and Pain, HAQ-DI, and FACIT-F outcomes at month 3 in csDMARD-IR patients. Proportions of csDMARD-IR patients at month 3 who did/did not report **a** mild Pain (VAS score ≤ 20 mm), **b** moderate improvements in Pain (≥ 30% decreases from baseline), **c** substantial improvements in Pain (≥ 50% decreases from baseline), **d** HAQ-DI scores ≥ normative values (≤ 0.25), or **e** FACIT-F scores ≥ normative values (≥ 43.5), stratified by LDA status (PtGA VAS score ≤ 20 mm). Denominators represent the number of patients who did/did not report LDA, respectively. Abbreviations: *BID* twice daily, *CI* confidence interval, *csDMARD* conventional synthetic disease-modifying antirheumatic drug, *FACIT-F* Functional Assessment of Chronic Illness Therapy-Fatigue, *HAQ-DI* Health Assessment Questionnaire-Disability Index, *IR* inadequate responder, *LDA* low disease activity, *PtGA* Patient Global Assessment of Disease Activity, *VAS* Visual Analog Scale

**Fig. 2:**
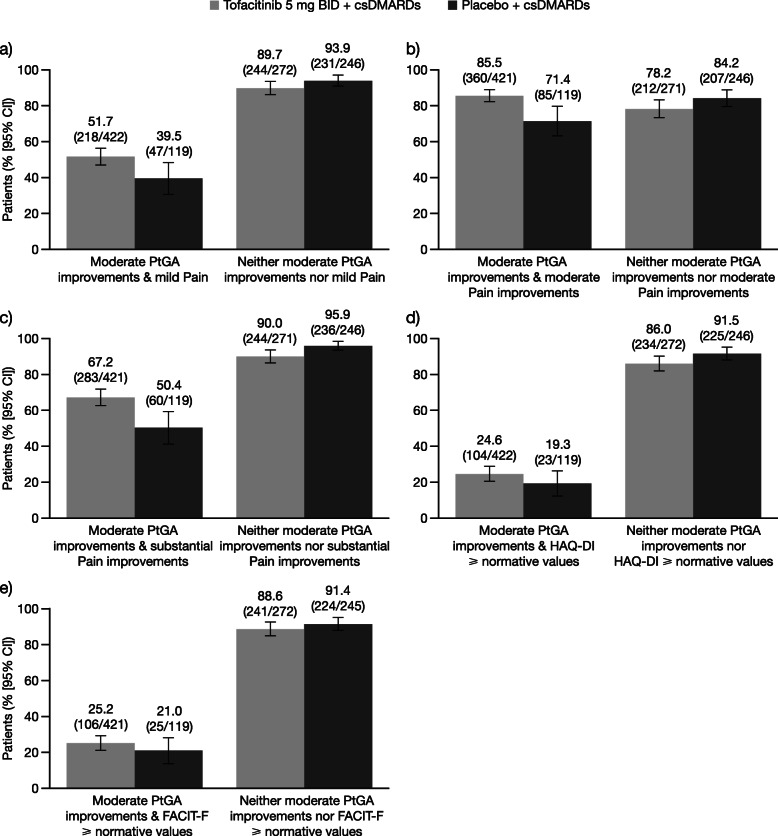
Associations between moderate improvements in PtGA and Pain, HAQ-DI, and FACIT-F outcomes at month 3 in csDMARD-IR patients. Proportions of csDMARD-IR patients at month 3 who did/did not report **a** mild Pain (VAS score ≤ 20 mm), **b** moderate improvements in Pain (≥ 30% decreases from baseline), **c** substantial improvements in Pain (≥ 50% decreases from baseline), **d** HAQ-DI scores ≥ normative values (≤ 0.25), or **e** FACIT-F scores ≥ normative values (≥ 43.5), stratified by reporting of moderate improvements in PtGA (≥ 30% decreases from baseline). Denominators represent the number of patients who did/did not report moderate PtGA improvements, respectively. Abbreviations: *BID* twice daily, *CI* confidence interval, *csDMARD* conventional synthetic disease-modifying antirheumatic drug, *FACIT-F* Functional Assessment of Chronic Illness Therapy-Fatigue, *HAQ-DI* Health Assessment Questionnaire-Disability Index, *IR* inadequate responder, *PtGA* Patient Global Assessment of Disease Activity, *VAS* Visual Analog Scale

**Fig. 3:**
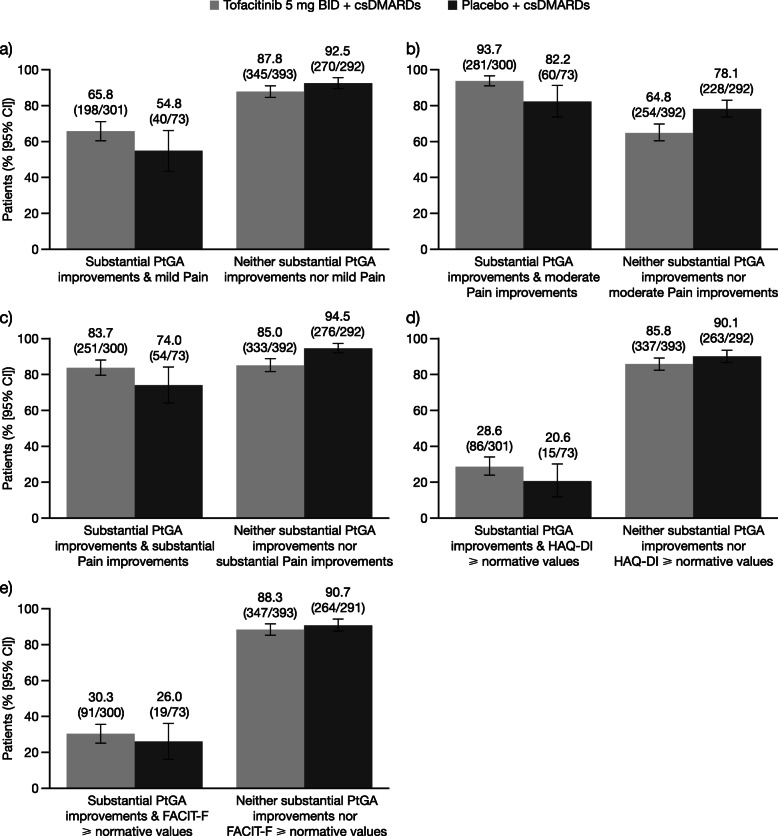
Associations between substantial improvements in PtGA and Pain, HAQ-DI, and FACIT-F outcomes at month 3 in csDMARD-IR patients. Proportions of csDMARD-IR patients at month 3 who did/did not report **a** mild Pain (VAS score ≤ 20 mm), **b** moderate improvements in Pain (≥ 30% decreases from baseline), **c** substantial improvements in Pain (≥ 50% decreases from baseline), **d** HAQ-DI scores ≥ normative values (≤ 0.25), or **e** FACIT-F scores ≥ normative values (≥ 43.5), stratified by reporting of substantial improvements in PtGA (≥ 50% decreases from baseline). Denominators represent the number of patients who did/did not report substantial PtGA improvements, respectively. Abbreviations: *BID* twice daily, *CI* confidence interval, *csDMARD* conventional synthetic disease-modifying antirheumatic drug, *FACIT-F* Functional Assessment of Chronic Illness Therapy-Fatigue, *HAQ-DI* Health Assessment Questionnaire-Disability Index, *IR* inadequate responder, *PtGA* Patient Global Assessment of Disease Activity, *VAS* Visual Analog Scale

At month 3, the proportion of csDMARD-IR patients reporting PtGA-defined LDA (VAS score ≤ 20 mm) with either mild pain (VAS score ≤ 20 mm) or moderate (≥ 30%) or substantial (≥ 50%) improvements in pain ranged from 86.0 to 91.6% in patients receiving tofacitinib and from 69.4 to 80.7% in patients receiving placebo. Similarly, a substantial proportion of patients receiving tofacitinib (53.4 to 88.3%) or placebo (75.6 to 95.4%) reported neither LDA nor any of the three Pain outcomes (Fig. 1a–c). In general, similar trends were evident when data were stratified by moderate (≥ 30% decreases from baseline; Fig. 2a–c) and substantial (≥ 50% decreases from baseline; Fig. 3a–c) improvements in PtGA. An exception to this was observed with moderate PtGA improvements and mild Pain: numerically lower proportions of patients receiving tofacitinib (51.7%) and placebo (39.5%) reported both of these outcomes compared with the other PtGA and Pain outcomes. The proportions of patients reporting each outcome alone are presented in Supplementary Figs. 1a–c to 3a–c (see Additional file 1).

In comparison with analyses of LDA and Pain outcomes, lower proportions of csDMARD-IR patients reported both LDA and scores ≥ normative values in either HAQ-DI (≤ 0.25) or FACIT-F (≥ 43.5) in the tofacitinib (37.0% and 40.5%, respectively) and placebo (29.0% and 33.9%, respectively) groups (Fig. 1d–e). In line with analyses of LDA and Pain outcomes, a substantial proportion of patients reported neither LDA nor normative values in either HAQ-DI or FACIT-F in the tofacitinib (87.1% and 89.4%, respectively) and placebo (91.5% and 91.8%, respectively) groups. These trends were also evident when data were stratified by moderate (Fig. 2d–e) and substantial PtGA improvements (Fig. 3d–e). The proportions of patients reporting each outcome alone are presented in Supplementary Figs. 1–3d and f (see Additional file 1).

In comparison with analyses of LDA and Pain outcomes, lower proportions (although still a majority) of csDMARD-IR patients reported both LDA and improvements from baseline ≥ MCID in either HAQ-DI (≥ 0.22) or FACIT-F (≥ 4.0) in the tofacitinib (79.6% and 65.0%, respectively) and placebo (61.3% and 47.5%, respectively) groups (Supplementary Fig. 4 in Additional file 1). Similarly, compared with analyses of LDA and Pain outcomes, lower proportions of patients reported neither LDA nor improvements from baseline ≥ MCID in either HAQ-DI or FACIT-F in the tofacitinib (39.0% and 49.5%, respectively) and placebo (57.6% and 66.2%, respectively) groups. In general, similar trends were seen when data were stratified by moderate and substantial PtGA improvements (Supplementary Figs. 5–6 in Additional file 1). The proportions of csDMARD-IR patients reporting each outcome alone are presented in Supplementary Figs. 1–3e and g (see Additional file 1).

In general, Figs. 1, 2, and 3 had the expected symmetrical appearance, with the tofacitinib-treatment group showing a higher proportion of csDMARD-IR patients meeting the criteria for both PtGA and Pain/HAQ-DI/FACIT-F outcomes versus the placebo group, and the placebo-treatment group showing a higher proportion of csDMARD-IR patients who met neither criterion versus the tofacitinib group.

Similar trends were observed in csDMARD-naïve and bDMARD-IR patients in the supporting analysis (i.e., the general order of strength of association with PtGA was pain > disability; Supplementary Figs. 7a–d to 8a–d [see Additional file 1]).

### Correlations between PtGA and pain, PtGA and HAQ-DI, and PtGA and FACIT-F

Correlation analyses show that in general, Pain was moderately-to-strongly associated with PtGA at month 3 in csDMARD-IR patients, irrespective of treatment group (correlation coefficient generally > 0.5), with the strongest correlation evident between LDA and mild Pain (Table 3). Pain was also moderately-to-strongly associated with PtGA at month 3 in csDMARD-naïve and bDMARD-IR patients (correlation coefficient generally > 0.5; Supplementary Table 2 [see Additional file 1]).

**Table 3:** Pearson Phi correlations between PtGA and Pain, HAQ-DI, and FACIT-F outcomes at month 3 in csDMARD-IR patients

	*** N***	Correlation coefficient	*P* value
**a) Correlation with LDA (PtGA VAS score ≤ 20 mm)**			
**Mild Pain (VAS score ≤ 20 mm)**			
Tofacitinib 5 mg BID + csDMARDs	695	0.74	< 0.0001
Placebo + csDMARDs	366	0.73	< 0.0001
**Moderate Pain improvement (≥ 30% decrease from baseline)**			
Tofacitinib 5 mg BID + csDMARDs	692	0.42	< 0.0001
Placebo + csDMARDs	365	0.45	< 0.0001
**Substantial Pain improvement (≥ 50% decrease from baseline)**			
Tofacitinib 5 mg BID + csDMARDs	692	0.55	< 0.0001
Placebo + csDMARDs	365	0.58	< 0.0001
**HAQ-DI score ≥ normative value (≤ 0.25)**			
Tofacitinib 5 mg BID + csDMARDs	695	0.28	< 0.0001
Placebo + csDMARDs	366	0.24	< 0.0001
**HAQ-DI change ≥ MCID (≥ 0.22 improvement from baseline)**			
Tofacitinib 5 mg BID + csDMARDs	693	0.18	< 0.0001
Placebo + csDMARDs	364	0.14	0.0064
**FACIT-F score ≥ normative value (≥ 43.5)**			
Tofacitinib 5 mg BID + csDMARDs	694	0.35	< 0.0001
Placebo + csDMARDs	365	0.29	< 0.0001
**FACIT-F change ≥ MCID (≥ 4.0 improvement from baseline)**			
Tofacitinib 5 mg BID + csDMARDs	693	0.13	0.0004
Placebo + csDMARDs	363	0.11	0.0413
**b) Correlation with moderate PtGA improvement (≥ 30% decrease from baseline)**			
**Mild Pain (VAS score ≤ 20 mm)**			
Tofacitinib 5 mg BID + csDMARDs	694	0.42	< 0.0001
Placebo + csDMARDs	365	0.42	< 0.0001
**Moderate Pain improvement (≥ 30% decrease from baseline)**			
Tofacitinib 5 mg BID + csDMARDs	692	0.64	< 0.0001
Placebo + csDMARDs	365	0.55	< 0.0001
**Substantial Pain improvement (≥ 50% decrease from baseline)**			
Tofacitinib 5 mg BID + csDMARDs	692	0.56	< 0.0001
Placebo + csDMARDs	365	0.55	< 0.0001
**HAQ-DI score ≥ normative value (≤ 0.25)**			
Tofacitinib 5 mg BID + csDMARDs	694	0.13	0.0006
Placebo + csDMARDs	365	0.16	0.0029
**HAQ-DI change ≥ MCID (≥ 0.22 improvement from baseline)**			
Tofacitinib 5 mg BID + csDMARDs	692	0.30	< 0.0001
Placebo + csDMARDs	364	0.30	< 0.0001
**FACIT-F score ≥ normative value (≥ 43.5)**			
Tofacitinib 5 mg BID + csDMARDs	693	0.17	< 0.0001
Placebo + csDMARDs	364	0.18	0.0008
**FACIT-F change ≥ MCID (≥ 4.0 improvement from baseline)**			
Tofacitinib 5 mg BID + csDMARDs	692	0.24	< 0.0001
Placebo + csDMARDs	363	0.26	< 0.0001
**c) Correlation with substantial PtGA improvement (≥ 50% decrease from baseline)**			
**Mild Pain (VAS score ≤ 20 mm)**			
Tofacitinib 5 mg BID + csDMARDs	694	0.55	< 0.0001
Placebo + csDMARDs	365	0.50	< 0.0001
**Moderate Pain improvement (≥ 30% decrease from baseline)**			
Tofacitinib 5 mg BID + csDMARDs	692	0.59	< 0.0001
Placebo + csDMARDs	365	0.51	< 0.0001
**Substantial Pain improvement (≥ 50% decrease from baseline)**			
Tofacitinib 5 mg BID + csDMARDs	692	0.68	< 0.0001
Placebo + csDMARDs	365	0.70	< 0.0001
**HAQ-DI score ≥ normative value (≤ 0.25)**			
Tofacitinib 5 mg BID + csDMARDs	694	0.18	< 0.0001
Placebo + csDMARDs	365	0.13	0.0126
**HAQ-DI change ≥ MCID (≥ 0.22 improvement from baseline)**			
Tofacitinib 5 mg BID + csDMARDs	692	0.29	< 0.0001
Placebo + csDMARDs	364	0.26	< 0.0001
**FACIT-F score ≥ normative value (≥ 43.5)**			
Tofacitinib 5 mg BID + csDMARDs	693	0.23	< 0.0001
Placebo + csDMARDs	364	0.20	0.0001
**FACIT-F change ≥ MCID (≥ 4.0 improvement from baseline)**			
Tofacitinib 5 mg BID + csDMARDs	692	0.26	< 0.0001
Placebo + csDMARDs	363	0.23	< 0.0001

Generally, correlation coefficient values around 0.3, 0.5, and 0.7 are considered as weak, moderate, and strong positive linear correlations, respectively

Abbreviations: *BID* twice daily, *csDMARD* conventional synthetic disease-modifying antirheumatic drug, *FACIT-F* Functional Assessment of Chronic Illness Therapy-Fatigue, *HAQ-DI* Health Assessment Questionnaire-Disability Index, *IR* inadequate responder, *LDA* low disease activity, *MCID* minimum clinically important difference, *PtGA* Patient Global Assessment of Disease Activity, *VAS* Visual Analog Scale

In contrast, across both treatment arms, HAQ-DI and FACIT-F were generally weakly correlated with PtGA at month 3 in csDMARD-IR patients (correlation coefficient <  0.3). In csDMARD-naïve and bDMARD-IR patients, HAQ-DI was weakly-to-moderately associated with PtGA at month 3 in tofacitinib-treated patients (correlation coefficient > 0.3 to < 0.5).

## Discussion

This post hoc analysis used pooled data from csDMARD-IR RA patients enrolled in three phase 3 RCTs of tofacitinib to examine the degree to which PtGA is driven by patient-reported improvements in pain, physical function, and fatigue. This is the first such analysis to be conducted in a tofacitinib-treated population of csDMARD-IR RA patients, and, to the best of our knowledge, the first to explore the effect of tofacitinib on specific pain outcomes (≥ 30% and ≥ 50% improvements from baseline in pain scores and attainment of mild Pain) in RA patients. The multiple clinically meaningful PRO improvements used in this study may offer an advantage over direct anchoring measures, such as the Patient Acceptable Symptom State (PASS), as they allow the evaluation of changes in PROs that may not be associated with an acceptable state of “feeling well,” as defined by PASS. Furthermore, definitions of PASS are not universally accepted and may differ across patient populations.

The results of this analysis expand upon the previously reported improvements in PtGA, Pain, physical function, and fatigue in phase 3 [26–30] and phase 3b/4 trials [31] of tofacitinib, by exploring and associating low symptom state attainment with the reporting of moderate and substantial clinical PRO improvements. In this analysis, clinically meaningful improvements in both PtGA and Pain (≥ 30% or ≥ 50% decreases from baseline), as well as the attainment of LDA (assessed by PtGA), mild Pain and improvements ≥ MCID in physical function (HAQ-DI) and fatigue (FACIT-F) were reported by large proportions of csDMARD-IR patients treated with tofacitinib 5 mg BID. As expected, across all endpoints, responses were higher with active therapy versus placebo, demonstrating that treatment with tofacitinib 5 mg BID greatly relieves the burden of disease from a patient perspective, reflecting a broad clinical benefit in RA patients. With guidelines shifting towards a more patient-centered approach to care [51], better understanding of the impact of treatment on patient-reported disease activity may help establish an improved standard for therapy assessment and modification.

The findings of our analysis indicate that PtGA responses are closely associated with Pain in csDMARD-IR patients. In both tofacitinib- and placebo-treated patients, subgroup analyses from 2 × 2 tables showed that the reporting of mild Pain, and clinically meaningful improvements in Pain, were associated with improvements in PtGA and attainment of LDA. In addition, lack of Pain improvement was associated with little or no improvement in PtGA. Generally, across PtGA and Pain response definitions, lower proportions of patients reported either clinically meaningful improvements (≥ 30% or ≥ 50% decreases from baseline) or a normative PtGA score (LDA) or mild Pain alone, compared with those who reported both or neither outcomes. Separate analyses of csDMARD-naïve and bDMARD-IR cohorts found generally similar associations between PtGA LDA and Pain responses.

In contrast, associations between PtGA and HAQ-DI, and PtGA and FACIT-F were less clear. When evaluating the association between PtGA and HAQ-DI, large proportions of csDMARD-IR patients reported improvements in PtGA alone, suggesting that PtGA assessments were not contingent on reporting clinically meaningful improvements in HAQ-DI scores. Separate analyses found generally similar results for HAQ-DI in csDMARD-naïve and bDMARD-IR patients. Similar trends were evident with FACIT-F scores ≥ normative values, again indicating that PtGA improvements were less strongly associated with fatigue. Although some trends were observed with clinically meaningful improvements in HAQ-DI and FACIT-F, large proportions of patients reported improvements ≥ MCID in HAQ-DI and FACIT-F or PtGA improvements alone. Thus, it is challenging to draw clear conclusions regarding the association of either HAQ-DI or FACIT-F improvements with PtGA.

The greater associations observed between PtGA and Pain, compared with the other PROs were further supported by correlation analyses. Pearson Phi correlation coefficients at month 3 indicated a stronger association between PtGA and Pain than between PtGA and HAQ-DI or FACIT-F (either improvements ≥ MCID or scores ≥ normative values) in csDMARD-IR patients in the main analysis. In the supporting analysis, a stronger association between PtGA and Pain than between PtGA and HAQ-DI (scores ≥ normative values) was observed in csDMARD-naïve and bDMARD-IR patients. In line with previous evidence from other RA trials, these results indicate that Pain is the key driver of PtGA [9, 12–15]; while physical function and fatigue influence PtGA to a lesser extent [12, 14]. The results of this analysis are further corroborated by the findings of an international survey of 1958 RA patients, in which patients most frequently defined a “good day” as a day free of pain; interestingly, the majority of patients also characterized a “good day” as being free of fatigue, and, to a lesser extent, the ability to engage in all activities [52]. These observations support existing evidence that pain alleviation is particularly important to RA patients and that sensitivity and attention to pain are crucial in meeting patients’ expectations of their arthritis care [12, 52]. A previous study has shown that pain remains a primary priority for patients, regardless of overall improvements in health status following treatment [53]. Moreover, pain is the most common symptom experienced by RA patients [54] and is the primary reason why patients with inflammatory arthritis see a rheumatologist [15].

The importance of adequately addressing patients’ pain levels in parallel with monitoring broader disease activity is further emphasized by the fact that pain has been shown to persist in RA patients who had Disease Activity Score in 28 joints, CRP (DAS28-4[CRP]) < 2.6 for over 1 year [55]. In the same study, CRP, SJC, TJC, and Sharp scores were not found to be significantly associated with increased pain severity at baseline or 1 year, indicating a non-inflammatory pain component is at play [55]. This hypothesis is further supported by a recently published study, which reported that a substantial proportion of RA patients reported unacceptable pain levels despite inflammation control following 2 years of early active treatment (begun < 1 year following RA onset) [56]. It has been proposed that increased sensitivity and presence of non-inflammatory pain in RA patients is due to central nervous system alterations in pain processing [57, 58]. In line with this theory, decreased pain thresholds in RA patients have been reported in both inflamed joints and tissues unaffected by inflammation [57, 58]. These findings further emphasize the importance of regular assessment of PROs, particularly those which evaluate pain. Without these, disease activity goals such as remission per composite measures may be achieved, yet patients whose pain is not adequately monitored/controlled are significantly more likely to report that their treatment expectations remain unmet [59].

A 2015 analysis of PROs in practice reported that clinicians are often reluctant to use PROs routinely due to a fear that it will add to their workload, rather than improve their efficiency and effectiveness [60]; therefore, it is important that incorporation of PROs into clinical practice not be burdensome [61]. PtGA is a single question that takes little time to ask and requires no training to interpret, making it a feasible, efficient measure of disease activity in the clinical setting [62]. However, while the results of this analysis indicate that, captured alone, PtGA correlates strongly with Pain, the weak correlations seen between PtGA and HAQ-DI/FACIT-F demonstrate that it provides little insight into patients’ physical function or levels of fatigue. In contrast, RAPID-3 (Routine Assessment of Patient Index Data 3), a pooled index of PtGA, Pain, and HAQ-DI that equally weights all three components [63], is a quick and easy method to obtain a comprehensive overview of patient wellbeing that has been shown to correlate strongly with clinical measures of disease activity, such as DAS28-4, erythrocyte sedimentation rate and Clinical Disease Activity Index [64, 65]. Given prior research and our current findings, clinicians using RAPID-3 may also consider collecting a single additional measure, fatigue (e.g., FACIT-F or fatigue VAS), to gain a more robust picture of patient state in a time-pressured clinical setting.

Some limitations remain associated with this post hoc analysis. First, as placebo-treated patients were advanced to tofacitinib treatment at month 3 (non-responders only) or month 6 (all remaining placebo-treated patients), data in this post hoc analysis were only evaluated up to month 3, a relatively short period to adequately investigate the associations. In addition, interpretations of PtGA by patients will vary and depend on a range of additional factors, such as comorbidities, disease duration, and patient expectations [9]. Furthermore, as PtGA asks patients to assess their disease activity based on “all the ways your disease affects you,” their scoring is based on other impacts of disease that may not be queried by pain, physical function, or fatigue. There may also have been an impact of unmeasured, non-RA factors such as demographic characteristics, education level, and cultural or geographical influences on patient assessments and perceptions of disease activity and burden. Moreover, painful comorbid conditions not impacted by treatment with tofacitinib or csDMARDs may have influenced the pain scores recorded by study patients. However, it is plausible that any such effect would have been similar between groups due to the randomized trial design. While the Pearson Phi correlation coefficients are useful for measuring linear relationships, the correlation analysis conducted was exploratory in nature; therefore, results should be interpreted with caution. There is a potential bias in this analysis as the assessments of PtGA and pain were based on similar measurement scales (VAS). However, we are not aware of any literature that has established that PRO associations are solely or predominantly based on using the same measurement scale. Prior work has shown that pain is a major determinant of PtGA in RA, but this was not due to the measurement scales used (VAS or numerical rating scale) [9]. The current analysis found not only a similar association between improvements in Pain and PtGA, but also one (albeit weaker) between HAQ-DI and PtGA, despite HAQ-DI data being collected using different questions. As previously noted, prior research has found occasional discordance between PtGA and MDGA in patients with RA [12, 13, 17, 19], even when those outcomes were collected via similar scales [17]. Finally, patient numbers in the csDMARD-naïve and bDMARD-IR cohorts were low, compared with the csDMARD-IR cohort, and further study will be required to confirm the results of this analysis in these patient populations.

## Conclusions

For the first time, this post hoc analysis of pooled data from three phase 3 RCTs of csDMARD-IR RA patients demonstrates the associations between PtGA and pain, physical function, and fatigue in tofacitinib-treated patients, corroborating the importance of clinically meaningful improvements in PROs and attainment of LDA states for the optimization of patient care. Similar findings were generally seen in csDMARD-naïve and bDMARD-IR patients. Overall, the findings support the importance of PtGA in clinical practice, and the role of Pain, and, to a lesser extent, physical function and fatigue, in driving patients’ perceptions of disease activity. While PtGA remains one of the most widely reported PROs, RAPID-3 represents a time-efficient approach that collects three outcomes (PtGA, Pain, and HAQ-DI) and may be supplemented by the addition of a single fatigue measure to provide a more robust picture of patient wellbeing.

## Supplementary information

**Additional file 1: Supplementary Tables 1–2; Supplementary Figures 1–8.** Proportions of patients who did/did not report improvements in HAQ-DI and FACIT-F scores ≥ MCID, stratified by clinically meaningful PtGA improvements; proportions of patients who reported clinically meaningful PtGA improvements or clinically meaningful Pain, HAQ-DI, or FACIT-F improvements alone; and Pearson Phi correlations between PtGA, and Pain and HAQ-DI outcomes.

## Data Availability

Upon request, and subject to certain criteria, conditions, and exceptions, see (https://www.pfizer.com/science/clinical-trials/trial-data-and-results for more information), Pfizer will provide access to individual de-identified participant data from Pfizer-sponsored global interventional clinical studies conducted for medicines, vaccines, and medical devices (1) for indications that have been approved in the USA and/or EU or (2) in programs that have been terminated (i.e., development for all indications has been discontinued). Pfizer will also consider requests for the protocol, data dictionary, and statistical analysis plan. Data may be requested from Pfizer trials 24 months after study completion. The de-identified participant data will be made available to researchers whose proposals meet the research criteria and other conditions, and for which an exception does not apply, via a secure portal. To gain access, data requestors must enter into a data access agreement with Pfizer.

## Abbreviations

ACR: American College of Rheumatology
bDMARD: Biologic disease-modifying antirheumatic drug
BID: Twice daily
CI: Confidence interval
CRP: C-reactive protein
csDMARD: Conventional synthetic disease-modifying antirheumatic drug
DAS28–4: Disease Activity Score in 28 joints
ESR: Erythrocyte sedimentation rate
EULAR: European League Against Rheumatism
FACIT-F: Functional Assessment of Chronic Illness Therapy-Fatigue
HAQ-DI: Health Assessment Questionnaire-Disability Index
HRQoL: Health-related quality of life
IR: Inadequate responder
LDA: Low disease activity
LTE: Long-term extension
MCID: Minimum clinically important difference
MDGA: Physician Global Assessment of Arthritis
MR: Modified-release
MTX: Methotrexate
OMERACT: Outcome Measures in Rheumatology
PASS: Patient Acceptable Symptom State
PRO: Patient-reported outcome
PtGA: Patient Global Assessment of Disease Activity
QD: Once daily
RA: Rheumatoid arthritis
RAPID-3: Routine Assessment of Patient Index Data 3
RCT: Randomized controlled trial
RF: Rheumatoid factor
SD: Standard deviation
SF-36: 36-Item Short Form Health Survey
SJC: Swollen joint count
TJC: Tender joint count
VAS: Visual Analog Scale
