# Supplementary material for: Associations between Patient Global Assessment scores and pain, physical function, and fatigue in rheumatoid arthritis: a post hoc analysis of data from phase 3 trials of tofacitinib
Source: Arthritis Res Ther. 2020 Oct 15;22:243. doi: 10.1186/s13075-020-02324-7 (PMC7566034; doi:10.1186/s13075-020-02324-7)
Supplement: Supplementary file 1 — Additional file 1: Supplementary Tables 1–2; Supplementary Figures 1–8. Proportions of patients who did/did not report improvements in HAQ-DI and FACIT-F scores ≥ MCID, stratified by clinically meaningful PtGA improvements; proportions of patients who reported clinically meaningful PtGA improvements or clinically meaningful Pain, HAQ-DI, or FACIT-F improvements alone; and Pearson Phi correlations between PtGA, and Pain and HAQ-DI outcomes. [file 13075_2020_2324_MOESM1_ESM.docx]

**Additional file 1**

**Supplementary Table 1** Proportion of csDMARD-naïve^a^ and bDMARD-IR^b^ patients reporting each clinically meaningful PRO improvement at month 3

|  | **csDMARD-naïve population^a^** | | **bDMARD-IR population^b^** | |
| --- | --- | --- | --- | --- |
| **PRO, *n* (%)** | **Tofacitinib 5 mg BID monotherapy**  **(*N* = 356)^c^** | **MTX monotherapy**  **(*N* = 173)^c^** | **Tofacitinib 5 mg BID + MTX**  **(*N* = 119)^c^** | **Placebo + MTX**  **(*N* = 118)^c^** |
| PtGA |  |  |  |  |
| LDA (PtGA VAS score ≤20 mm) | 140 (39.3) | 39 (22.5) | 34 (28.6) | 14 (11.9) |
| Substantial PtGA improvement (≥50% decrease from baseline) | 162 (45.5) | 54 (31.2) | 45 (37.8) | 21 (17.8) |
| Pain |  |  |  |  |
| Mild Pain (VAS score ≤20 mm) | 147 (41.3) | 43 (24.9) | 40 (33.6) | 13 (11.0) |
| Substantial Pain improvement (≥50% decrease from baseline) | 178 (50.0) | 62 (35.8) | 52 (43.7) | 19 (16.1) |
| HAQ-DI |  |  |  |  |
| HAQ-DI score ≥ normative value (≤0.25) | 108 (30.3) | 24 (13.9) | 19 (16.0) | 11 (9.3) |

^a^Includes patients from ORAL Start (NCT01039688) [40]
^b^Includes patients from ORAL Step (NCT00960440) [41]
^c^Patients with an ACR20 response at Month 3.
Abbreviations: *ACR20* 20% improvement in American College of Rheumatology criteria, *bDMARD* biologic disease-modifying antirheumatic drug, *BID* twice daily, *csDMARD* conventional synthetic disease-modifying antirheumatic drug, *HAQ-DI* Health Assessment Questionnaire-Disability Index, *IR* inadequate response, *LDA* low disease activity, *MTX* methotrexate, *PRO* patient-reported outcome, *PtGA* Patient Global Assessment of Disease Activity, *VAS* Visual Analog Scale

**Supplementary Table 2** Pearson Phi correlations between PtGA, and Pain and HAQ‑DI outcomes at month 3 in csDMARD-naïve^a^ and bDMARD-IR^b^ patients

|  | 1. **Correlation with LDA  (PtGA VAS score ≤20 mm)** | | |
| --- | --- | --- | --- |
|  | ***N*** | Correlation coefficient | *P* value |
| **Mild Pain (VAS score ≤20 mm)** | | | |
| csDMARD-naïve patients^a^ |  |  |  |
| Tofacitinib 5 mg BID monotherapy | 349 | 0.78 | < 0.0001 |
| MTX monotherapy | 170 | 0.71 | < 0.0001 |
| bDMARD-IR patients^b^ |  |  |  |
| Tofacitinib 5 mg BID + MTX | 119 | 0.77 | < 0.0001 |
| Placebo + MTX | 116 | 0.62 | < 0.0001 |
| **Substantial Pain improvement (≥50% decrease from baseline)** | | | |
| csDMARD-naïve patients^a^ |  |  |  |
| Tofacitinib 5 mg BID monotherapy | 348 | 0.51 | < 0.0001 |
| MTX monotherapy | 170 | 0.55 | < 0.0001 |
| bDMARD-IR patients^b^ |  |  |  |
| Tofacitinib 5 mg BID + MTX | 114 | 0.66 | < 0.0001 |
| Placebo + MTX | 115 | 0.41 | < 0.0001 |
| **HAQ-DI score ≥normative value (≤0.25)** | | | |
| csDMARD-naïve patients^a^ |  |  |  |
| Tofacitinib 5 mg BID monotherapy | 351 | 0.41 | < 0.0001 |
| MTX monotherapy | 169 | 0.18 | 0.0195 |
| bDMARD-IR patients^b^ |  |  |  |
| Tofacitinib 5 mg BID + MTX | 118 | 0.33 | 0.0002 |
| Placebo + MTX | 118 | 0.33 | 0.0002 |

|  | 1. **Correlation with substantial  PtGA improvement  (≥50% decrease from baseline)** | | |
| --- | --- | --- | --- |
|  | *N* | Correlation coefficient | *P* value |
| **Mild Pain (VAS score ≤20 mm)** | | | |
| csDMARD-naïve patients^a^ |  |  |  |
| Tofacitinib 5 mg BID monotherapy | 349 | 0.61 | < 0.0001 |
| MTX monotherapy | 365 | 0.49 | < 0.0001 |
| bDMARD-IR patients^b^ |  |  |  |
| Tofacitinib 5 mg BID + MTX | 119 | 0.70 | < 0.0001 |
| Placebo + MTX | 116 | 0.65 | < 0.0001 |

Generally, correlation coefficient values around 0.3, 0.5, and 0.7 are considered as weak, moderate, and strong positive linear correlations, respectively
Abbreviations: *bDMARD* biologic disease-modifying antirheumatic drug, *BID* twice daily, *csDMARD* conventional synthetic disease-modifying antirheumatic drug, *HAQ-DI* Health Assessment Questionnaire-Disability Index, *IR* inadequate responder, *LDA* low disease activity, *MTX* methotrexate, *PtGA* Patient Global Assessment of Disease Activity, *VAS* Visual Analog Scale

**Supplementary Fig. 1** Proportions of csDMARD-IR patients at month 3 who reported either LDA (PtGA VAS score ≤20 mm) or a) mild Pain (VAS score ≤20 mm), b) moderate improvements in Pain (≥30% decreases from baseline), c) substantial improvements in Pain (≥50% decreases from baseline), d) HAQ-DI scores ≥normative values (≤0.25), e) improvements in HAQ-DI scores ≥ MCID (≥0.22 improvement from baseline), f) FACIT-F scores ≥normative values (≥43.5), or g) improvements in FACIT-F scores ≥ MCID (≥4 improvement from baseline) alone


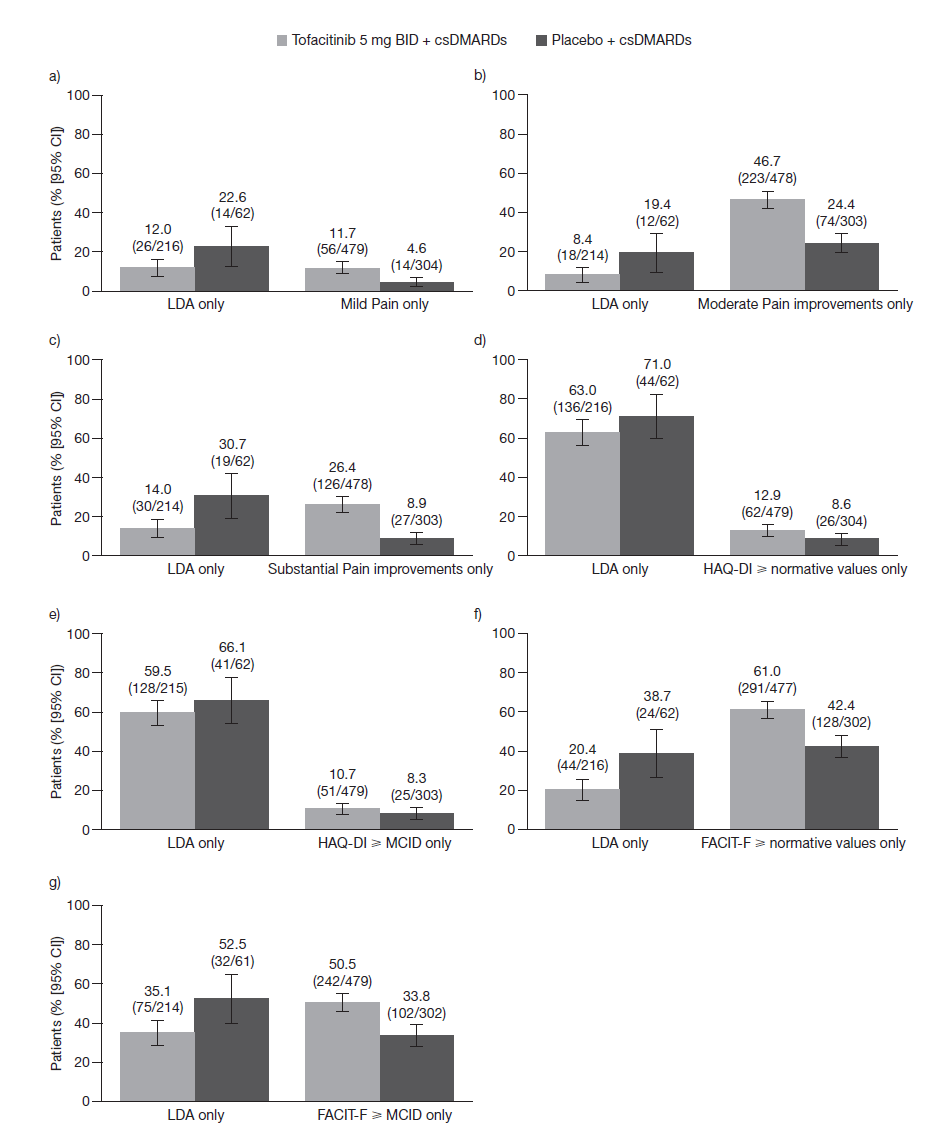


Denominators represent the number of patients who did/did not report LDA
Abbreviations: *BID* twice daily, *CI* confidence interval, *csDMARD* conventional synthetic disease‑modifying antirheumatic drug, *FACIT-F* Functional Assessment of Chronic Illness Therapy-Fatigue, *HAQ-DI* Health Assessment Questionnaire-Disability Index, *IR* inadequate responder, *LDA* low disease activity, *MCID* minimum clinically important difference, *PtGA* Patient Global Assessment of Disease Activity, *VAS* Visual Analog Scale

**Supplementary Fig. 2** Proportions of csDMARD-IR patients at month 3 who reported either moderate improvements in PtGA (≥30% decreases from baseline in PtGA) or a) mild Pain (VAS score ≤20 mm), b) moderate improvements in Pain (≥30% decreases from baseline), c) substantial improvements in Pain (≥50% decreases from baseline), d) HAQ-DI scores ≥normative values (≤0.25), e) improvements in HAQ-DI scores ≥ MCID (≥0.22 improvement from baseline), f) FACIT-F scores ≥normative values (≥43.5), or g) improvements in FACIT-F scores ≥ MCID (≥4 improvement from baseline) alone


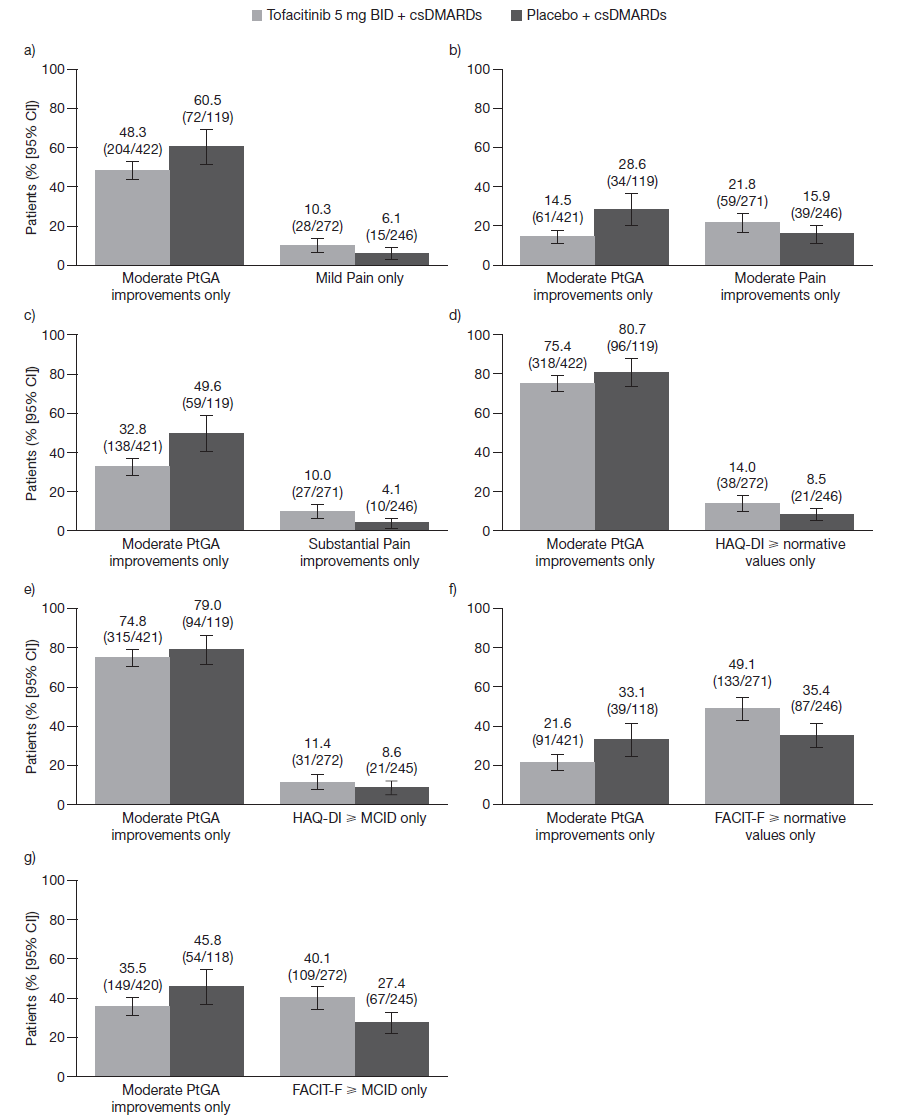


Denominators represent the number of patients who did/did not report moderate PtGA improvements
Abbreviations: *BID* twice daily, *CI* confidence interval, *csDMARD* conventional synthetic disease-modifying antirheumatic drug, *FACIT-F* Functional Assessment of Chronic Illness Therapy-Fatigue, *HAQ-DI* Health Assessment Questionnaire-Disability Index, *IR* inadequate responder, *MCID* minimum clinically important difference, *PtGA* Patient Global Assessment of Disease Activity, *VAS* Visual Analog Scale

**Supplementary Fig. 3** Proportions of csDMARD-IR patients at month 3 who achieved substantial improvements in PtGA (≥50% decreases from baseline in PtGA) or a) mild Pain (VAS score ≤20 mm), b) moderate improvements in Pain (≥30% decreases from baseline), c) substantial improvements in Pain (≥50% decreases from baseline), d) HAQ-DI scores ≥normative values (≤0.25), e) improvements in HAQ-DI scores ≥ MCID (≥0.22 improvement from baseline), f) FACIT-F scores ≥normative values (≥43.5), or g) improvements in FACIT-F scores ≥ MCID (≥4 improvement from baseline) alone


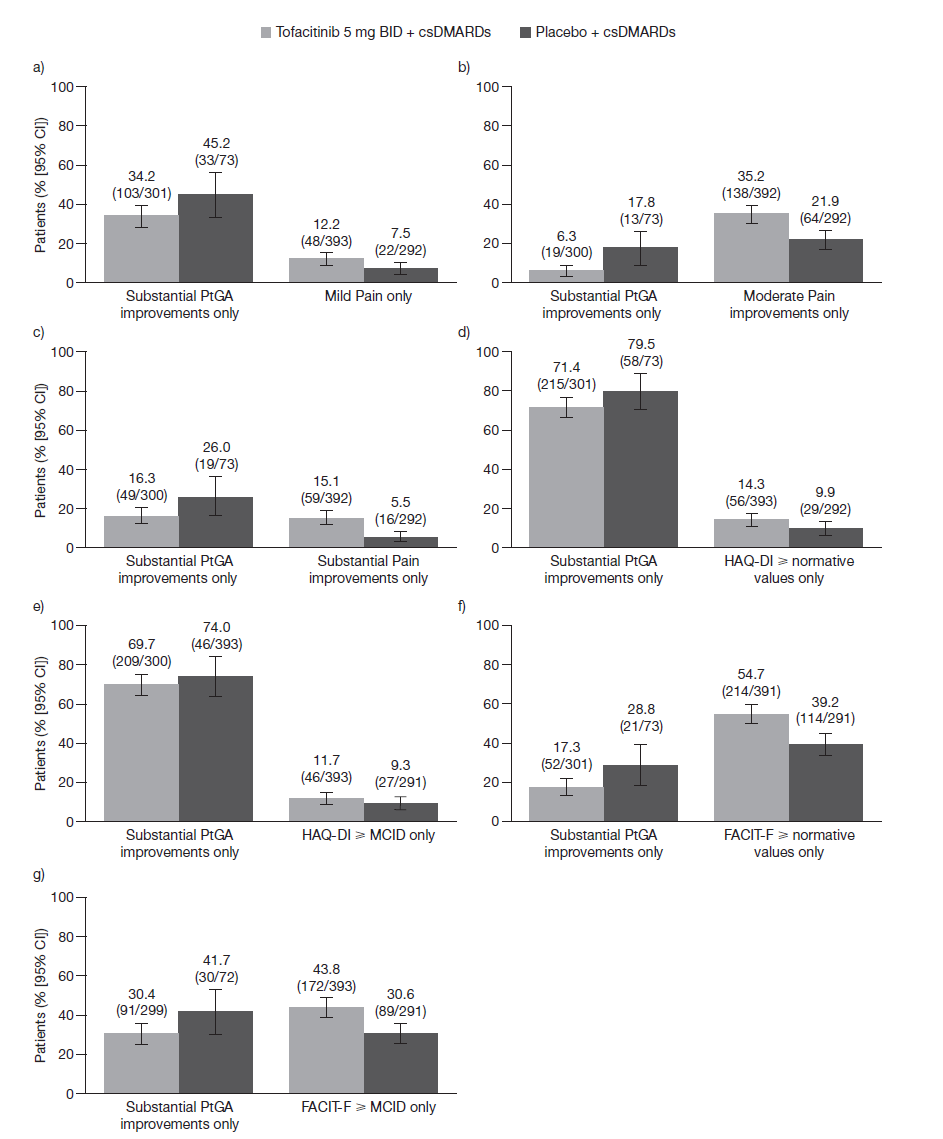


Denominators represent the number of patients who did/did not report substantial PtGA improvements
Abbreviations: *BID* twice daily, *CI* confidence interval, *csDMARD* conventional synthetic disease-modifying antirheumatic drug, *FACIT-F* Functional Assessment of Chronic Illness Therapy-Fatigue, *HAQ-DI* Health Assessment Questionnaire-Disability Index, *IR* inadequate responder, *MCID* minimum clinically important difference, *PtGA* Patient Global Assessment of Disease Activity, *VAS* Visual Analog Scale

**Supplementary Fig. 4** Proportions of csDMARD-IR patients at month 3 who did/did not report a) improvements in HAQ-DI scores ≥ MCID (≥0.22 improvement from baseline), or b) improvements in FACIT-F scores ≥ MCID (≥4 improvement from baseline), stratified by LDA status (PtGA VAS score ≤20 mm)


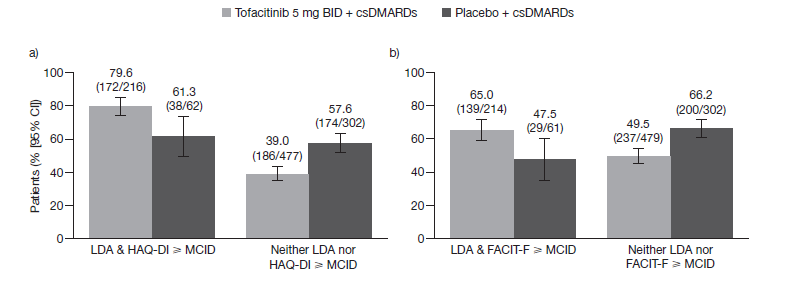
Denominators represent the number of patients who did/did not report LDA, respectively
Abbreviations: *BID* twice daily, *CI* confidence interval, *csDMARD* conventional synthetic disease-modifying antirheumatic drug, *FACIT-F* Functional Assessment of Chronic Illness Therapy-Fatigue, *HAQ-DI* Health Assessment Questionnaire-Disability Index, *LDA* low disease activity, *IR* inadequate responder, *MCID* minimum clinically important difference, *PtGA* Patient Global Assessment of Disease Activity, *VAS* Visual Analog Scale

**Supplementary Fig. 5** Proportions of csDMARD-IR patients at month 3 who did/did not report a) improvements in HAQ-DI scores ≥ MCID (≥0.22 improvement from baseline), or b) improvements in FACIT-F scores ≥ MCID (≥4 improvement from baseline), stratified by reporting of moderate improvements in PtGA (≥30% decreases from baseline)


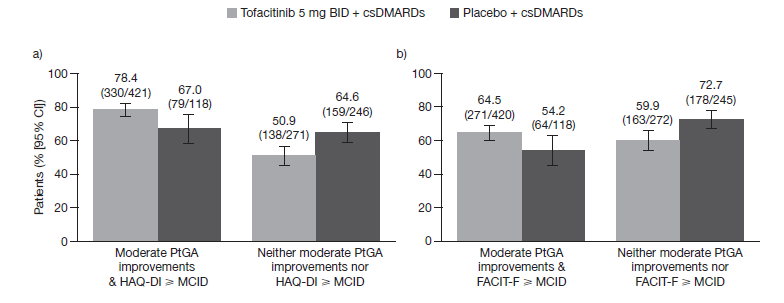
Denominators represent the number of patients who did/did not report moderate PtGA improvements, respectively
Abbreviations: *BID* twice daily, *CI* confidence interval, *csDMARD* conventional synthetic disease-modifying antirheumatic drug, *FACIT-F* Functional Assessment of Chronic Illness Therapy-Fatigue, *HAQ-DI* Health Assessment Questionnaire-Disability Index, *IR* inadequate responder, *MCID* minimum clinically important difference, *PtGA* Patient Global Assessment of Disease Activity

**Supplementary Fig. 6** Proportions of csDMARD-IR patients at month 3 who did/did not report a) improvements in HAQ-DI scores ≥ MCID (≥0.22 improvement from baseline), or b) improvements in FACIT-F scores ≥ MCID (≥4 improvement from baseline), stratified by reporting of substantial improvements in PtGA (≥50% decreases from baseline)


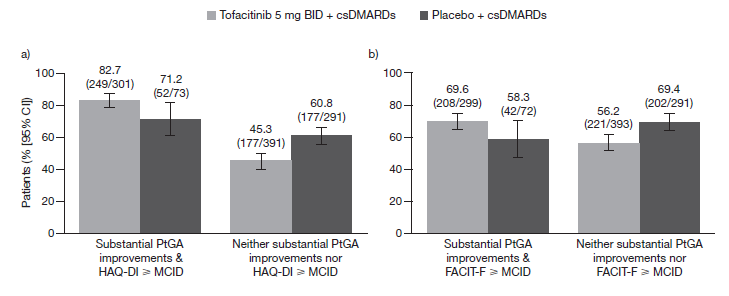
Denominators represent the number of patients who did/did not report substantial PtGA improvements
Abbreviations: *BID* twice daily, *CI* confidence interval, *csDMARD* conventional synthetic disease-modifying antirheumatic drug, *FACIT-F* Functional Assessment of Chronic Illness Therapy-Fatigue, *HAQ-DI* Health Assessment Questionnaire-Disability Index, *IR* inadequate responder, *MCID* minimum clinically important difference, *PtGA* Patient Global Assessment of Disease Activity

**Supplementary Fig. 7** Proportions of csDMARD-naïve patients at month 3 who reported a) LDA (PtGA VAS score ≤20 mm) or mild Pain (VAS score ≤20 mm), b) LDA (PtGA VAS score ≤20 mm) or substantial improvements in Pain (≥50% decreases from baseline), c) LDA (PtGA VAS score ≤20 mm) or HAQ-DI scores ≥normative values (≤0.25), or d) substantial improvements in PtGA (≥50% decreases from baseline) or mild Pain (VAS score ≤20 mm)


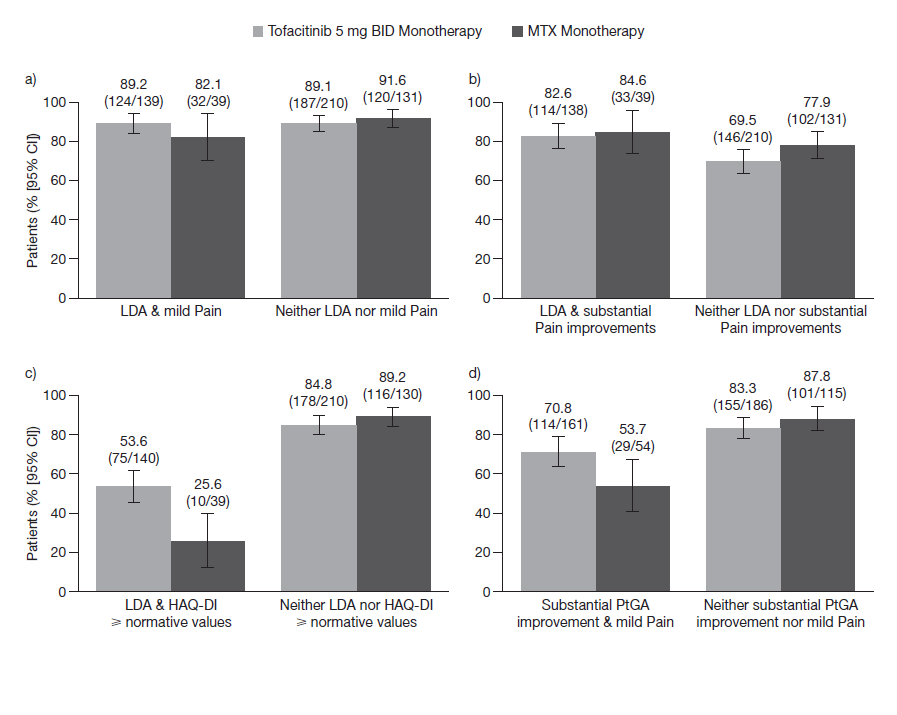


In panels a–c, denominators represent the number of patients who did/did not report LDA; in panel d, denominators represent the number of patients who did/did not report substantial PtGA improvements
Abbreviations: *BID* twice daily, *CI* confidence interval, *csDMARD* conventional synthetic disease-modifying antirheumatic drug, *HAQ-DI* Health Assessment Questionnaire-Disability Index, *LDA* low disease activity, *MTX* methotrexate, *PtGA* Patient Global Assessment of Disease Activity, *VAS* Visual Analog Scale

**Supplementary Fig. 8** Proportions of bDMARD-IR patients at month 3 who reported a) LDA (PtGA VAS score ≤20 mm) or mild Pain (VAS score ≤20 mm), b) LDA (PtGA VAS score ≤20 mm) or substantial improvements in Pain (≥50% decreases from baseline), c) LDA (PtGA VAS score ≤20 mm) or HAQ-DI scores ≥normative values (≤0.25), or d) substantial improvements in PtGA (≥50% decreases from baseline) or mild Pain (VAS score ≤20 mm)


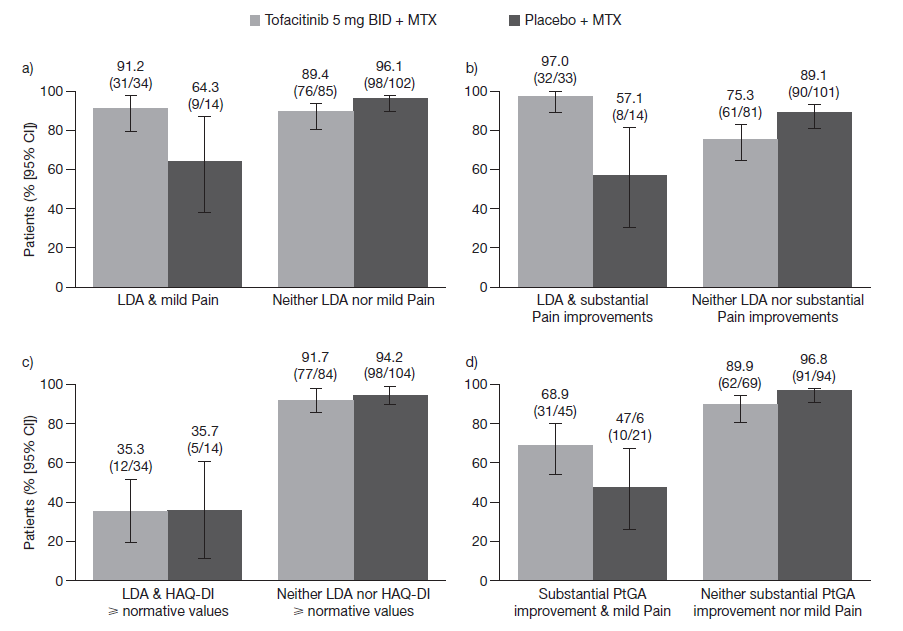


In panels a–c, denominators represent the number of patients who did/did not report LDA; in panel d, denominators represent the number of patients who did/did not report substantial PtGA improvements
Abbreviations: *bDMARD* biologic disease-modifying antirheumatic drug, *BID* twice daily, *CI*confidence interval, *HAQ-DI* Health Assessment Questionnaire-Disability Index, *IR* inadequate responder, *LDA* low disease activity, *MTX* methotrexate, *PtGA* Patient Global Assessment of Disease Activity, *VAS* Visual Analog Scale
